# A Study on the Relationship Between Consumer Motivations and Subjective Well-Being: A Latent Profile Analysis

**DOI:** 10.3389/fpsyg.2022.938060

**Published:** 2022-06-23

**Authors:** Jun Xiao, Yanping Gong, Jian Li, Xiuyuan Tang, Sohail Ahmad Javeed, Yuling Deng

**Affiliations:** ^1^School of Business, Central South University, Changsha, China; ^2^School of Management, Hunan City University, Yiyang, China; ^3^School of Business, Anhui University, Hefei, China; ^4^School of Business Administration, Hunan University of Finance and Economics, Changsha, China; ^5^Health Management Center, The Third Xiangya Hospital, Central South University, Changsha, China

**Keywords:** consumer motivations, subjective well-being, person-centered approach, latent profile analysis, positive affect, negative affect

## Abstract

Consumer motivation plays an important role in their purchase decisions and well-being. Previous studies have investigated the relationship between certain consumer motivations and well-being separately. We aim to employ latent profile analysis (LPA) to explore subgroups of participants who display similar patterns of consumer multiple motivations and to examine differences in subjective well-being across these subtypes. The final group of (*N* = 1,023, ages 18–58) completed the Consumer Motivation scale online, assessing seven dimensions of consumer motivation. Results of LPA identified four subpopulations of participants: the enthusiastic group has high expectations in all aspects; the balanced grou*p* values each aspect of the product but has lower expectations; the rational group emphasizes aspects such as value for money, comfort, and quality; the apathetic group has no strong motivation for consumption, and they are relatively concerned with the dimensions of security, social acceptance, and stimulation. Consumers’ subjective well-being (life satisfaction, positive affect, and negative affect) differed significantly across the four profiles. Specifically, scores for positive affect and life satisfaction were highest in the enthusiastic group, medium in the balanced group, and lowest in the rational and apathetic groups. Scores for negative affect were significantly higher in the enthusiastic and apathetic groups than in the rational group. These findings enable marketers to develop customized marketing strategies for different motivation profiles and contribute to helping consumers with varying motivation profiles to consciously pay attention to their well-being.

## Introduction

The pursuit of happiness is the fundamental impetus for human beings. Many consumers view consumption as a way to improve their quality of life (Chang, 2020; An et al., 2021). It is crucial for companies as well, not only in relation to customer loyalty (Troebs et al., 2018) and positive word-of-mouth (El Hedhli et al., 2016; Kim et al., 2018), but also for long-term relationships with customers. Visionary companies should consider their consumers’ well-being an important strategic objective and a responsibility for success in an increasingly competitive marketplace.

A growing number of scholars are focusing on what contributes to consumers’ happiness (Chang, 2020; An et al., 2021), and some studies have preliminarily noticed that consumer motivations have an effect on their well-being (Akram et al., 2021a). For example, consumption of hedonistic products leads to increased life satisfaction and subjective well-being (Zhong and Mitchell, 2010). High-ethical consumers expect positive emotions from their sustainable consumption (Onwezen et al., 2013). However, other studies have shown that not all consumer motivations predict well-being positively (e.g., Oral and Thurner, 2019; Shahid and Paul, 2021). Conspicuous consumption driven by the desire to show off possessions and status may make them less happy (Shahid and Paul, 2021). Materialism has been shown to be associated with lower levels of life satisfaction (Gong et al., 2020b), as motivated by the pursuit of too much consumption and acquisition (Oral and Thurner, 2019). A number of motivations have been examined individually in previous studies, but little is known about their combined effect on consumer well-being.

In fact, consumer motivations do not exist in isolation. In the consumers’ purchasing selection process, multiple motivation dimensions are co-activated, and these activated motivations often involve complex relationships. Various consumer motivations may support or conflict with each other in influencing consumers’ consumption. For instance, when buying pro-environmental products, consumers might have a range of motivations from ethics to saving money, comfort, and excitement, and they always have to weigh these motivations (Moisander, 2007; Steg et al., 2014). Pro-environmental behavior may be consistent with consumers’ ethical motivations but not compatible with their economic and hedonic motivations (Steg et al., 2014; Rezvani et al., 2018). Other research has shown that personal moral and gaining motivation can enhance positive anticipated emotions (hedonic motivation) when adopting an electric vehicle (Rezvani et al., 2018).

While it is acknowledged that consumers may have multiple and sometimes conflicting motivations for a given consumer behavior (Steg et al., 2014; Akram et al., 2021b), most of the previous studies ignored the complex connections among motivations. Moreover, individuals’ motivation to purchase is subjective (Moisander, 2007; Steg et al., 2014; Gong et al., 2020a). Different groups have varying perceptions of the importance of the dimensions of consumer motivation (Kim et al., 2014; Singh, 2018). An empirical understanding of how these motivations combine and affect consumer well-being is limited. The lack of this information is due in part to a reliance on a variable-centered approach to research. The variable-centered approach treats the sample as a homogeneous group, assuming a certain motivation as one of many factors motivating an individual’s affect and behaviors (Morin and Marsh, 2014; Morin et al., 2016). The results obtained by this approach aim to exert an effect on the outcome by adding one motivation without considering other motivations that may simultaneously affect the outcome, and this omission may affect the effectiveness of interventions (Morin and Marsh, 2014).

By contrast, the person-centered approach can shed light on the interaction among multiple consumer psychological motivations in making consumption decisions by segregating consumers’ subtypes with similar consumer motivation patterns (Howard et al., 2016; Gong et al., 2020a; Xiao and Sun, 2020). The person-centered approach of latent profile analysis (LPA) is a model-based classification method that can provide more objective subtype identification criteria and lower classification errors (Howard et al., 2016; Gong et al., 2020a). To fulfill this research gap, the primary objective of this study was to reanalyze the relationship between consumer motivation and subjective well-being by employing a person-centered approach. Specifically, we employed the LPA technique, which provided an ideal method to capture distinct patterns of consumer motivation and their complex relationship with well-being.

Taken together, there are three main objectives of this study. First, by applying the person-centered approach of the LPA method and using an integrative measurement, we explored different consumer subgroups exhibiting similar patterns of consumer motivations. Second, there may be differences in the demographic characteristics of different customer profile subtypes, and we make comparisons. Third, the present research tested the possibility that consumers’ subjective well-being varies according to these subgroup profiles.

The current study presents a nuanced extension of the literature related to consumer motivation and well-being. From a holistic perspective, understanding consumer motivation profiles is a means to better understand the role of consumer motivation in predicting consumers’ subjective well-being. In addition, our findings can effectively help consumers with different motivation patterns to understand the changes in their well-being and enable companies to develop distinct marketing strategies to promote sales based on the purchase motivations of different market segments.

The paper is structured as follows: Section “Literature Review” reviews the literature on the dimensions of consumer motivations and their relationships with subjective well-being. Section “Materials and Methods” explains the research method, including survey procedures and measures for all constructs referenced in this study. A detailed explanation of the research results in section “Results” follows the discussion in section “Discussion”, which outlines the implications, limitations, and suggestions for further research. The conclusion is presented in section “Conclusion.”

## Literature Review

### Multi-Dimensionality of Consumer Motivation

As consumers, our motivations represent our needs, which influence our perception and choice of products (Kim et al., 2014; Singh, 2018). Diverse consumer motivations have been considered one of the most important predictors of consumer purchase decisions (Akram et al., 2021b). Identifying consumer motivations can provide the basis for understanding consumer needs and market segmentation.

There are different views on the structure of consumer motivation (e.g., Babin et al., 1994; Arnold and Reynolds, 2003; Lindenberg and Steg, 2007). The research of Babin et al. (1994) developed and tested a hedonic-utilitarian shopping value model. Arnold and Reynolds (2003) created a measurement of hedonic motivation, comprised of six dimensions: adventure, social, gratification, idea, role, and value shopping. In their study, Arnold and Reynolds (2003) created a measure of hedonic motivation that encompasses six dimensions: adventure, social, gratification, idea, role, and value shopping. To guide environmental behavior, Lindenberg and Steg (2007) developed the goal-framing theory (hedonic goals, gain goals, and normative goals), and the utilitarian and hedonic motivational frameworks have been extended to three motivation frames.

More recently, based on the goal-framing theory (Lindenberg and Steg, 2007), Barbopoulos and Johansson (2017) developed the Consumer Motivation Scale (CMS), which assesses three first-order motivations—gain, hedonic, and normative—and their seven dimensions. Specifically, the gain motivation is sensitive to changes in individual resources and includes three dimensions: value for money (pay a reasonable price and avoid wasting money), quality (buy something of high quality or useful), and safety (feel calm and prepared for the unforeseen). Hedonic motivation is sensitive to changes in one’s pleasure and mood and includes two dimensions: stimulation (getting something exciting, stimulating, or unique; avoid dullness); and comfort (buying something pleasant and comfortable; avoid hassle and discomfort). The normative motivation focuses on the appropriateness of the act and is sensitive to what “should” be (Lindenberg and Steg, 2007; Barbopoulos and Johansson, 2016, 2017). As concerns about environmental issues and conspicuous consumption rise (Steg et al., 2014; Shahid and Paul, 2021), normative motivation is further conceptualized as internal and external normative motivations, referred to as ethics and social acceptance, respectively. While ethics emphasizes acting in accordance with one’s moral principles and obligations and avoiding guilt, social acceptance implies making a good impression and living up to the expectations of others. Moreover, the CMS is a valuable measure because it assesses a wide variety of consumer contexts, including products (clothing, food, and housing), and services (entertainment, and travel).

These factors of consumer motivation are not separate from each other. A few empirical studies explored the interactions between the motivation factors with a variable-centered approach. For example, Rezvani et al. (2018) found that gain, hedonic, and normative motivations have direct positive effects on consumers’ purchase of electric vehicles, but gain and normative motivations can also strengthen hedonic motivations and indirectly influence purchase intent. Johansson et al. (2020) have shown that when social and moral motivations (social acceptance and ethics) are co-activated, people become less value-conscious (value for money) for costly green consumption because in this way they appear to be moral in the eyes of others.

Prior studies on the structure of consumer motivations provide a solid theoretical basis for typologies of consumer motivation. Several countries have segmented consumer groups based on different structures of consumer motivations (see Table 1). Ribeiro Cardoso and Carvalho Pinto (2010) found five groups among Portuguese adult consumers: social shoppers, dynamic shoppers, rational shoppers, moderate shoppers, and involved shoppers. Based on the two dimensions of convenience and entertainment, Gilboa and Vilnai-Yavetz (2012) classified Israeli consumers into three categories: enthusiasts, recreational, and utilitarians. Kim et al. (2014) integrated motivations for hedonic benefits, utilitarian benefits, and shopping costs to segment consumers under the American retail context into four categories: involved shoppers; rational shoppers; experiential shoppers; and nonchalant shoppers. In a study of Turkish shopping center consumers, Kabadayi and Paksoy (2016) identified four consumer types: serious consumers; recreational consumers; enthusiastic consumers; and rational consumers. Singh (2018) integrated materialism into hedonic (Arnold and Reynolds, 2003) and utilitarian (Babin et al., 1994) motivations and segmented Indian mall shoppers into four segments: balanced shoppers, materialists, hedonists, and value seekers. This research has provided useful insights into the typology of consumer motivation.

**Table 1:** A review of recent consumer motivations typologies studies across countries.

Study (Year)	Context (Country)	Methods	Identified motivations	Shopping typologies (Features)
Ribeiro Cardoso and Carvalho Pinto (2010)	Shopping (Portugal)	Cluster analysis	Hedonic (Pleasure and gratification, Idea shopping, Social shopping, Role shopping, Value shopping); Utilitarian (Achievement, Efficiency)	Social shopper, Dynamic shopper, Rational shopper, Moderate shopper, Involved shopper
Gilboa and Vilnai-Yavetz (2012)	Malls (Israel)	Cluster analysis	Convenience, Entertainment	Enthusiasts, Recreationals, Utilitarians
Kim et al. (2014)	Retail setting (USA)	Cluster analysis	Utilitarian (Accessibility, Price, Convenience); Hedonic (Image, Ambience, Enjoyment); Shopping Cost (Search, Transportation, Psychic)	Involved shoppers, Rational shoppers, Experiential shoppers, Nonchalant shoppers
Kabadayi and Paksoy (2016)	Shopping center (Turkey)	Cluster analysis	Experiential, Goal-oriented, Socializing, Time saving, Deal-seeking	Serious consumers, Recreational consumers, Enthusiastic consumers, Rational consumers
Singh (2018)	Shopping mall (Indian)	Cluster analysis	Hedonic (Adventure/Gratification shopping, Role shopping, Idea shopping, Value shopping, Social shopping) Utilitarian; Materialism	Balanced shoppers, Materialist shoppers, Hedonistic shoppers, Value shoppers

These studies, described in Table 1, have advanced our understanding of consumer behavior by highlighting different profiles or subgroups of consumers. However, these studies focus exclusively on hedonic and utilitarian aspects of consumer motivation without considering normative aspects (i.e., ethics and social acceptance). They used cluster analysis, and it is more advisable to use LPA than cluster analysis in studying taxonomy (Howard et al., 2016; Li et al., 2021).

Latent profile analysis and cluster analysis are commonly used person-centered methods for identifying homogenous consumer subpopulations. Traditional cluster analysis is considered to be too sensitive to clustering algorithms and metric scales and relies on strict assumptions (e.g., accurate assignment of each sample to a single profile; Howard et al., 2016). By contrast, LPA is a more flexible and model-based classification method by allowing the estimation of alternative models and accordingly relaxing the restrictive conditions of cluster analysis (Howard et al., 2016). Importantly, LPA can provide more objective classification indicators for selecting the optimal number of profiles, with reasonable subtype identification criteria and lower classification errors (Wang and Hanges, 2010; Li et al., 2021). The profiles identified by LPA are typical in nature and referred to as potential. Instead of forcing each individual to correspond to a single profile, it assigns all participants a probability of becoming a profile based on the similarity to each typical potential profile (Howard et al., 2016). There have been several studies using the LPA approach to segment consumers, but these studies have focused on specific scenarios such as motivations for food choice (Gong et al., 2020a), ethical consumption (Shim and Cho, 2021), and tourism (Song and Bae, 2017). No study to date has used the LPA method in studying consumer motivation.

Additionally, most extant typology studies were conducted in traditional physical shopping contexts, such as a shopping center or mall where data were collected through mall intercept (e.g., Mehta et al., 2013; Singh, 2018). This study focuses on understanding the motivational characteristics of a wide range of consumers in different regions of Chinese consumers, with data collected online. Furthermore, with changes in business models during the last two decades, coupled with the recent COVID-19 outbreak, brick-and-mortar department stores are suffering a decline in sales (Hou and Elliott, 2021). As more and more consumers flock to online shopping (Akram et al., 2020), collecting data online is a better way for a more general consumer motivation as well as a broader sample. Besides, in order to highlight the differences in consumer motivation for different products, our survey set up five consumption contexts.

Therefore, in light of theoretical and empirical considerations, this study used the new technique of LPA and the CMS to identify profiles of consumers. Since this study employs a new dimensional structure of consumer motivation under the Chinese context, it is preferable to use exploratory LPA rather than posing specific hypotheses.

### The Relationship of Consumer Motivation and Well-Being

Previous literature has examined the association between well-being-relevant outcomes and the seven dimensions of consumer motivation separately to varying degrees. According to Barbopoulos and Johansson (2017), the gain goal was identified as three distinct dimensions: value for money, quality, and security. In everyday life, the concept of value for money is understood as the notion that a good or service is not paid for more than its quality or availability (Glendinning, 1988). Perceived value is often closely related to quality, and when the price of the product rises, consumers tend to expect that the quality should be higher (Dodds et al., 1991; Barbopoulos and Johansson, 2016). Perceived value and perceived quality are important predictors of customer satisfaction (Samudro et al., 2020). The dimension of security is related to the pursuit of harmony and stability and the avoidance of financial security risks when purchasing, so it may be beneficial to long-term well-being (Barbopoulos and Johansson, 2017). However, insecurity and fear of the future have been found to be associated with lower psychological well-being (Callea et al., 2019).

Two dimensions of hedonic motivation have been verified: one is comfort, representing a state of physical and mental comfort, avoiding troubles; the other is stimulation, representing activities that produce arousal (Barbopoulos and Johansson, 2017). The motivations for comfort and stimulating choices appear to be contradictory (Bello and Etzel, 1985), but within a reasonable range of arousal, an individual may seek both comfort and stimulation (Ormel et al., 1999). Stimulation within a pleasant range and comfort are associated with physical health in a positive way (Ormel et al., 1999; Barbopoulos and Johansson, 2017). Further, a prolonged level of high stimulation becomes unpleasant and even detrimental, and it was suggested that the link between stimulation and well-being is an inverted U-shape (Ormel et al., 1999).

Normative motivation is composed of two distinct dimensions: social acceptance and ethics (Barbopoulos and Johansson, 2017). Both dimensions are normative, meaning they involve acting appropriately by a certain standard (Lindenberg and Steg, 2007; Barbopoulos and Johansson, 2016), but they differ in the source of the standard. Social acceptance is external in origin, with the desire to conform to the expectations of others, and is associated with susceptibility to interpersonal influences (Barbopoulos and Johansson, 2017). According to Barbopoulos and Johansson (2017), ethics is a desire to behave in accordance with one’s values, moral principles, and obligations in order to avoid internal guilt (Barbopoulos and Johansson, 2017). Social and ethical norms also differ in their respective consequences. The former is associated with conspicuous or visible consumption (Barbopoulos and Johansson, 2017), whereas the latter is an important consideration in terms of pro-social behavior and ethical consumption (Lindenberg and Steg, 2007; Barbopoulos and Johansson, 2017). Multiple studies suggest that when people have high ambitions for extrinsic life goals for which social acceptance is an important factor, they may exhibit lower life satisfaction, self-esteem, and self-actualization, and higher depression and anxiety (Schmuck et al., 2000; Kasser and Ryan, 2016). Compared to extrinsic goals, the pursuit of intrinsic goals is associated with positive outcomes such as well-being and adjustment (Schmuck et al., 2000; Kasser and Ryan, 2016). Meanwhile, other results have also indicated that ethical motivation can induce people to engage in pro-environmental behavior (Onwezen et al., 2013), and pro-social behavior may be an effective way to promote happiness (Hui, 2022).

These results suggest that various dimensions of motivation explain different aspects of well-being. The dimensions of comfort (Ormel et al., 1999) and ethics (Hwang and Kim, 2018) appear to be positively associated with well-being; stimulation and well-being may have a U-shaped association (Hebb, 1958; Wippler and Lindenberg, 1987); and social acceptance may be negatively associated with well-being (Schmuck et al., 2000; Kasser and Ryan, 2016). The multi-dimensional nature of consumer motivation entails a more complex link to well-being than might be evident in the extant research. Thus, to understand the relationship between consumers’ multiple motivations and subjective well-being from a holistic perspective, it is essential to explore the presence of distinct subtypes with similar patterns of consumer motivation profiles based on their combined responses to these dimensions (value for money, quality, safety, stimulation, comfort, ethics, and social acceptance). It is difficult to solve this problem with the traditional variable-centered approach, and we address the question using a person-centered approach (Morin et al., 2016; Xiao and Sun, 2020). Considering the complex relationship between various consumer motivations and well-being and the fact that there has been no research on consumer motivations grouped according to the probabilistic theory, we conducted exploratory analyses using LPA rather than posing hypotheses.

## Materials and Methods

### Procedures and Sample

Respondents completed a self-administered questionnaire to collect data. To obtain a wide range of participants, the questionnaire was published through a professional online survey platform, Wenjuanxing.^1^ Subjects nationwide were selected to complete the online survey through a random sampling method. Two parts of the questionnaire were included: (a) Personal information about respondents: gender, age, education, disposable income, and occupation; and (b) Measures of consumer motivation and subjective well-being. By excluding respondents who could not answer two filter questions or who took more than 150 s to fill out the questionnaire, we eliminated respondents who were not serious. Participants were only allowed to enter their IP addresses once to prevent reentry. Over 1800 questionnaires were received in a month (September 2021). The final sample consisted of 1,023 participants from China, 66% of whom were women. The subjects were aged in the range of 18–58 years, with an average age of 29.94 years old. About half of the participants were married (50.2%). The majority of participants had a bachelor’s degree (62%), 17% of respondents had less than a university degree, and 21% of respondents had a graduate degree or above. More than half of the participants had a monthly disposable income < ¥5,000 (58%), 17.1% had a monthly disposable income of ¥5,000–8,000, and 24.9% had a monthly disposable income of ¥8,000 or more.

### Measures

#### Consumer Motivation

We adopted the CMS developed by Barbopoulos and Johansson (2017) to measure participants’ ratings of the dimensions of consumer motivation. The statement presented by CMS was “When I shop for ___, it is important that I choose.” The blanks were randomly assigned to one of five consumption scenarios (food, clothes, entertainment, or something fun, travel, and housing). Next, participants rated the importance of each statement of 34 items in their respective context, on a 7-point scale from 1 (not at all important) to 7 (extremely important). In the current study, Cronbach’s alphas for the seven dimensions of value for money, quality, safety, stimulation, comfort, ethics, and social acceptance were 0.87, 0.88, 0.89, 0.86, 0.79, 0.85, and 0.91, respectively.

#### Consumers’ Subjective Well-Being

As in earlier studies (Chang, 2020), subjective well-being was measured in terms of its cognitive component (life satisfaction) and its affective component (positive and negative affect). We utilized the Satisfaction with Life Scale (SWLS) to measure life satisfaction (Diener et al., 1985). The SWLS consists of five items, including “Most of the time, I am living close to my ideal.” Each item was scored on a 7-point Likert scale (1 = completely disagree to 7 = completely agree). In the current study, Cronbach’s alpha for the SWLS was 0.92.

We used the revised version of the Positive and Negative Affect Scale (PANAS) to assess affective experiences (Watson et al., 1988; Qiu et al., 2008). This scale is composed of nine positive affect words (e.g., “full of enthusiasm”) and nine negative affect words (e.g., “nervous”). Participants rated each item on a 5-point Likert scale (1 = very slight or not at all, 5 = strongly). In the current study, Cronbach’s alphas for positive and negative affect were 0.95 and 0.93, respectively.

The original English-language scales (i.e., CMS and SWLS) in this study were translated into Chinese using a translation (English to Chinese) and back-translation (Chinese to English) procedure. The translation and back-translation were further discussed by two professionals to ensure accuracy.

## Results

### Descriptive Statistics and Correlation Analyses

Table 2 shows the correlation coefficients and descriptive statistics of the focal variables in this study. The results showed low to moderate positive correlations among the seven consumer motivations (0.22 ≤ *r* ≤ 0.62). The positive affect and life satisfaction of consumers’ subjective well-being were positively correlated with each consumer motivation (0.15 ≤ *r* ≤ 0.50). However, the negative affect dimension of subjective well-being was not significantly correlated with value for money, quality, comfort, or ethics. These results provided a preliminary basis for identifying different consumer motivation groups and testing differences in subjective well-being across different groups.

**Table 2:** Descriptive statistics and correlation analyses.

Variables	M	SD	1	2	3	4	5	6	7	8	9	10
1. Value for money	6.02	0.75	**0.76**									
2. Quality	5.72	0.90	0.46^***^	**0.79**								
3. Safety	5.70	1.05	0.28^***^	0.47^***^	**0.79**							
4. Stimulation	5.03	1.05	0.27^***^	0.46^***^	0.38^***^	**0.74**						
5. Comfort	5.86	0.77	0.62^***^	0.57^***^	0.39^***^	0.39^***^	**0.71**					
6. Ethics	6.04	0.77	0.48^***^	0.46^***^	0.41^***^	0.36^***^	0.49^***^	**0.75**				
7. Social acceptance	5.06	1.19	0.22^***^	0.37^***^	0.38^***^	0.50^***^	0.36^***^	0.32^***^	**0.81**			
8. Positive affect	3.22	0.95	0.23^***^	0.43^***^	0.37^***^	0.50^***^	0.38^***^	0.30^***^	0.41^***^	**0.84**		
9. Negative affect	1.80	0.82	−0.04	0.05	0.07^*^	0.13^***^	0.01	−0.02	0.13^***^	−0.1^***^	**0.78**	
10. Life satisfaction	4.53	1.33	0.15^***^	0.35^***^	0.31^***^	0.33^***^	0.27^***^	0.22^***^	0.34^***^	0.63^***^	0.01	**0.84**

*n = 1,023. The diagonal values in bold represent the square root of AVE*.

* *p < 0.05 and*

*** *p < 0.001*.

### Confirmatory Factor Analysis

The results of the confirmatory factor analysis showed that the ten-factor model (7 dimensions of consumer motivation and 3 dimensions of subjective well-being) had acceptable fit to the data (*χ*^2^ = 5188.76, *df* = 1,494, *χ*^2^/*df* = 3.47, CFI = 0.91, TLI = 0.91, SRMR = 0.052, RMSEA = 0.049), and the fit indices were significantly better than those of the single-factor model (*χ*^2^ = 27533.91, *df* = 1,539, *χ*^2^/*df* = 17.89, CFI = 0.38, TLI = 0.36, SRMR = 0.144, RMSEA = 0.128, ∆*χ*^2^ = 22345.15, ∆*df =* 45, *p* < 0.001). The results support the construct validity of the scale, and also indicate that there was not a serious problem of common method bias.

Convergent validity was tested with standardized factor loadings, composite reliability (CR), and average variance extracted (AVE). Table 3 shows that the standardized factor loadings for each item were greater than the recommended value of 0.5. The CR of the focal constructs ranged from 0.80 to 0.95, which exceeds the recommended value of 0.7. The minimum value of the AVE of all constructs was 0.5, which meets the recommended standard of 0.5 (Hair et al., 2010). These three indicators provide evidence that the measurements of all constructs had good convergent validity. The correlation coefficient of each construct was lower than the corresponding square root of AVE, indicating acceptable discriminant validity (Fornell and Larcker, 1981; see Table 3). In light of the above, the construct measures of consumer motivation and subjective well-being showed construct, convergent, and divergent validity.

**Table 3:** Reliability and convergent validity of the constructs.

Construct	Dimensions	Items	Loading	Cronbach’s alpha	CR	AVE
Consumer motivations	Value for money	VM1	0.74	0.87	0.87	0.57
		VM2	0.73			
		VM3	0.79			
		VM4	0.77			
		VM5	0.75			
	Quality	QU1	0.67	0.88	0.89	0.62
		QU2	0.77			
		QU3	0.84			
		QU4	0.85			
		QU5	0.80			
	Safety	SA1	0.86	0.89	0.89	0.62
		SA2	0.90			
		SA3	0.75			
		SA4	0.79			
	Stimulation	ST1	0.80	0.86	0.86	0.54
		ST2	0.81			
		ST3	0.73			
		ST4	0.65			
		ST5	0.68			
	Comfort	CO1	0.69	0.79	0.80	0.50
		CO2	0.68			
		CO3	0.74			
		CO4	0.71			
	Ethics	ET1	0.68	0.85	0.86	0.56
		ET2	0.52			
		ET3	0.84			
		ET4	0.87			
		ET5	0.76			
	Social acceptance	SO1	0.66	0.91	0.90	0.65
		SO2	0.72			
		SO3	0.81			
		SO4	0.93			
		SO5	0.89			
Subjective well-being	Positive affect	PE1	0.68	0.95	0.95	0.70
		PE 2	0.85			
		PE 3	0.89			
		PE 4	0.85			
		PE 5	0.89			
		PE 6	0.87			
		PE 7	0.84			
		PE 8	0.83			
		PE 9	0.79			
	Negative affect	NE1	0.77	0.93	0.93	0.61
		NE 2	0.77			
		NE 3	0.73			
		NE 4	0.84			
		NE6	0.79			
		NE7	0.88			
		NE8	0.70			
		NE9	0.66			
	Life satisfaction	LS1	0.85	0.92	0.92	0.71
		LS2	0.82			
		LS3	0.90			
		LS4	0.82			
		LS5	0.81			

*CR*, *Composite Reliability, AVE, Average Variance.*

### Latent Profile Analysis of Consumer Motivation

We used Mplus 7.0 to identify the optimal profile model for consumer motivation referring to Asparouhov and Muthen’s (2014) steps. First, the average score for each dimension of consumer motivation is normalized before performing the LPA. Second, the optimal model is determined according to these fit indices: Akaike Information Criterion (AIC), Bayesian Information Criterion (BIC), Sample-Size-Adjusted BIC (SSABIC), the Lo–Mendell–Rubin adjusted Likelihood Ratio Test (LMR-LRT), Bootstrap Likelihood Ratio Test (BLRT), Entropy, Sample proportion of the profile (Nylund et al., 2007). Lower AIC, BIC, and SSABIC values represent better and more parsimonious profile model fits. Significant LMR-LRT and BLRT values indicate that the k profile model is superior to the k-1 profile model. When the Entropy value is greater than 0.8, it indicates that the model accuracy is good, meanwhile, it is required to meet the sample proportion of each profile should not be less than 5% (Nylund et al., 2007). Finally, the optimal model should be supported by the theory and each profile should have substantive meaning.

The model fit indices are shown in Table 4. The 4-profile model was optimal. Compared with the 3-profile model, the 4-profile model had higher entropy, and significant LMR-LRT and BLRT values. However, the 5-profile model had lower AIC, BIC, and SSABIC values, and significant BLRT. In comparison with the 4-profile model, the LMR-LRT *p* value of the 5-profile model was 0.67, and the smallest sample proportion was 2.05%, which is below the acceptable criterion. Taken together, these results support the selection of the 4-profile model.

**Table 4:** Summary of fit indices for one- to five-profile models.

Number of profiles	AIC	BIC	SSABIC	Entropy	LMR-LRT (*p* value)	BLRT (*p* value)	Smallest profile %
1	20153.28	20222.31	20177.85	—	—	—	—
2	18565.99	18674.46	18604.58	0.79	<0.001	<0.001	42.42%
3	18058.81	18206.72	18111.44	0.79	0.014	<0.001	15.34%
4	17841.14	18028.50	17907.81	0.81	0.044	<0.001	9.78%
5	17719.49	17846.29	17800.19	0.81	0.670	<0.001	2.05%

*AIC*, *Akaike Information Criterion; BIC, Bayesian Information Criterion; SSABIC, Sample-Size-Adjusted BIC; LMR-LRT, the Lo–Mendell–Rubin adjusted Likelihood Ratio Test; BLRT, Bootstrap Likelihood Ratio Test*.

**Figure 1:**
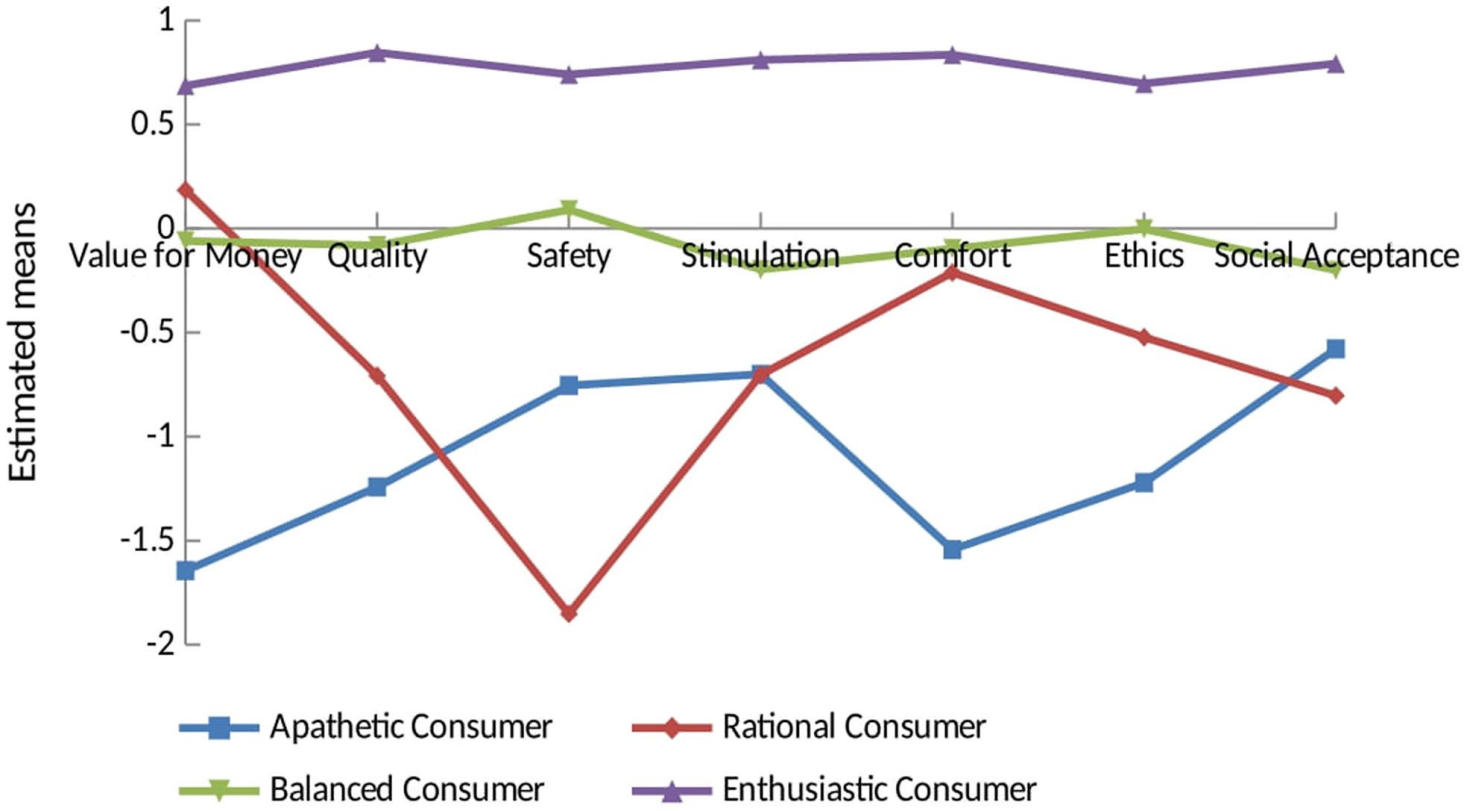
Latent profiles of different consumer motivation segment (*N* = 1023).

The 4-profile model also provided meaningful information about the full picture of consumer motivations. The raw means and estimates of consumer motivations for each profile are, respectively, shown in Table 5 and Figure 1. All profiles attached great importance to ethics and value for money. Participants with Profile 1 scored below average on all dimensions of consumer motivation, suggesting that consumers in this group lacked interest in consumption. Therefore, participants with Profile 1 were named Apathetic Consumers, containing 11.53% of consumers in this study (*n* = 118). Profile 2 was characterized by high scores for value for money, comfort, ethics, and quality. This group appeared to be very rational, and participants with this profile were labeled Rational Consumers. This profile represented the largest number of consumers in our sample, containing 9.78% of participants (*n* = 100). We called Profile 3 Balanced Consumers, as scores for each motive were near average. This was the largest profile in the current study, comprising 49.36% of the consumers (*n* = 505). Finally, Profile 4 is referred to as Enthusiastic Consumers in this study, with reference to Kabadayi and Paksoy (2016), as they scored highest on all motivational dimensions. This profile comprised 29.33% of the participants (*n* = 300).

**Table 5:** Average factor scores by the four profiles.

	Profile 1	Profile 2	Profile 3	Profile 4	*F*-test
	Apathetic consumer	Rational consumer	Balanced consumer	Enthusiastic consumer	
	*n* = 118 (11.53%)	*n* = 100 (9.78%)	*n* = 505 (49.36%)	*n* = 300 (29.33%)	
Value for money	4.71^d^ (3)	6.17^b^ (1)	5.97^c^ (2)	6.57^a^ (2)	350.54^***^
Quality	4.56^d^ (5)	5.05^c^ (4)	5.65^b^ (5)	6.51^a^ (5)	310.44^***^
Safety	4.92^c^ (2)	3.68^d^ (7)	5.80^b^ (3)	6.52^a^ (4)	522.11^***^
Stimulation	4.28^c^ (7)	4.24^c^ (5)	4.81^b^ (7)	5.94^a^ (7)	185.64^***^
Comfort	4.62^c^ (4)	5.67^b^ (2)	5.78^b^ (4)	6.54^a^ (3)	397.62^***^
Ethics	5.03^d^ (1)	5.57^c^ (3)	6.02^b^ (1)	6.62^a^ (1)	228.22^***^
Social acceptance	4.37^c^ (6)	4.06^c^ (6)	4.82^b^ (6)	6.08^a^ (6)	179.12^***^

*Figures in brackets refer to the ranking of factors based on mean scores. Different letters represent significant difference (p < 0.05)*.

*** *p < 0.001*.

### Differences in Demographic Variables Across Consumer Motivation Types

Cross-tabulation analysis and ANOVAs were used to examine whether demographic variables differed across the four profiles of consumer motivation (see Table 6). The results in Table 6 indicate that age [*F*_(3, 1,019)_ = 3.04, *p* < 0.05] and education [χ^2^_(9)_ = 63.52, *p* < 0.001] were significantly different across the consumer motivation profiles, but gender [*χ*^2^_(3)_ = 6.25, *p* > 0.05], income [*χ*^2^_(12)_ = 16.38, *p* > 0.05] and occupation [*χ*^2^_(24)_ = 27.05, *p* > 0.05] were not. More specifically, the average age of the enthusiastic consumer was 28.58 years old, skewed toward the young, whereas that of the balanced consumer was 30.53 years old, marginally older. The apathetic consumer group had a slightly higher percentage of members with a high school or lower education than the other three groups. Enthusiastic consumers tended to be more likely to have a college degree, while rational consumers were more likely to have a postgraduate degree or above. In addition, there were some differences in consumer contexts observed; the clothes consumption context had a higher proportion of rational consumers, the travel consumption context had a greater percentage of apathetic consumers, and most of the balanced consumers fell into the accommodations consumption context.

**Table 6:** Differences in demographic variables across the profiles (Cross-tab with Pearson Chi-square).

		1 Apathetic consumer *n* = 118	2 Rational consumer *n* = 100	3 Balanced consumer *n* = 505	4 Enthusiastic consumer *n* = 300	*χ*^2^-test (sig.)/*F*-test (sig.)	*p* Value
Gender	Male	45(38.1%)^a^	38(38.0%)^a^	213(42.2%)^a^	100(33.3%)^a^	*χ*^2^_(3)_ = 6.25	0.10
	Female	73(61.9%)^a^	62(62.0%)^a^	292(57.8%)^a^	200(66.7%)^a^		
Age		30.3^ab^	30.6^ab^	30.53^a^	28.58^b^	*F*_(3, 1,019)_ = 3.04^*^	0.03
Education	High school or lower	17(14.4%)^a^	4(4.0%)^ab^	48(9.5%)^ab^	19(6.3%)^b^	*χ*^2^_(9)_ = 63.52^***^	*p* < 0.001
	Junior college	8(6.8%)^ab^	3(3.0%)^b^	30(5.9%)^b^	44(14.7%)^a^		
	Bachelor degree	71(60.2%)^a^	52(52.0%)^a^	313(62.0%)^a^	199(66.3%)^a^		
	Postgraduate or above	22(18.6%)^ab^	41(41.0%)^a^	114(22.6%)^b^	38(12.7%)^c^		
Disposable income (1,000RMB/Month)	< 3	44(37.3%)^a^	35(35.0%)^a^	193(38.2%)^a^	113(37.7%)^a^	*χ*^2^_(12)_ = 16.38	0.17
	3–5	27(22.9%)^a^	16(16.0%)^a^	95(18.8%)^a^	70(23.3%)^a^		
	5–8	24(20.3%)^a^	22(22.0%)^a^	81(16.0%)^a^	48(16.0%)^a^		
	8–10	8 (6.8%)^a^	11(11.0%)^a^	51(10.1%)^a^	39(13.0%)^a^		
	>10	15(12.7%)^ab^	16(16.0%)^ab^	85(16.8%)^b^	30(10.0%)^a^		
Occupation	Civil servants or Staff of public institution	14(11.9%)^a^	10(10.0%)^a^	69(13.7%)^a^	46(15.3%)^a^	*χ*^2^_(24)_ = 27.05	0.30
	Professionals	9(7.6%)^a^	13(13.0%)^a^	61(12.1%)^a^	38(12.7%)^a^		
	White-collar worker	39(33.7%)^a^	31(31.0%)^a^	139(27.5%)^a^	83(27.7%)^a^		
	Service personnel	6(5.1%)^a^	2(2.0%)^a^	11(2.2%)^a^	6(2.0%)^a^		
	Free-lancers	2(1.7%)^ab^	5(5.0%)^ab^	22(4.4%)^b^	8(2.7%)^a^		
	Blue-collar workers	2(1.7%)^a^	3(3.0%)^a^	7(1.4%)^a^	10(3.3%)^a^		
	Students	36(30.5%)^a^	29(29.0%)^a^	154(30.5%)^a^	95(31.7%)^a^		
	Housewives	2(1.7%)^a^	1(1.0%)^a^	11(2.2%)^a^	9 (3.0%)^a^		
	Others	8(6.8%)^ab^	6(6.0%)^ab^	31(6.1%)^a^	5(1.7%)^b^		
Consumer contexts	Food	27(22.9%)^a^	24(24.0%)^a^	99(19.6%)^a^	61(20.3%)^a^	*χ*^2^_(12)_ = 45.21^***^	*p* < 0.001
	Clothes	21(17.8%)^b^	36(36.0%)^a^	83(16.4%)^b^	67(22.3%)^b^		
	Entertainment	25(21.2%)^a^	22(22.0%)^a^	113(22.4%)^a^	53(17.7%)^a^		
	Travel	31(26.3%)^a^	10(10.0%)^b^	89(17.6%)^ab^	65(21.7%)^ab^		
	Accommodations	14(11.9%)^ab^	8(8.0%)^b^	121(24.0%)^a^	54(18.0%)^ab^		

* *p < 0.05 and*

*** *p < 0.001*.

Different letters represent significant difference (p < 0.05).

### The Relationship Between Consumer Motivation Types and Subjective Well-Being

The ANOVA test was used to examine the relationship between the four consumer motivation profiles and subjective well-being. The results are presented in Table 7 showing that positive affect [*F*_(3, 1,019)_ = 111.74, *p* < 0.001], negative affect [*F*_(3, 1,019)_ = 5.71, *p* < 0.01] and life satisfaction [*F*_(3, 1,019)_ = 58.79, *p* < 0.001] vary significantly across the four profiles of consumer motivation. More specifically, pairwise comparative analysis of *Post hoc* Bonferroni tests showed that positive affect and life satisfaction were highest among the enthusiastic group, moderate for the balanced group, and lowest for the rational and apathetic groups. For negative affect, the rational group is significantly lower than the enthusiastic and apathetic groups, and the balanced group did not differ significantly from other groups.

**Table 7:** ANOVA of subjective well-being.

*n* = 1,023	Apathetic consumer	Rational consumer	Balanced consumer	Enthusiastic consumer	*F*
Positive affect	2.61^c^ (0.70)	2.56^c^ (0.83)	3.09^b^ (0.82)	3.88^a^ (0.88)	111.74^***^
Negative affect	1.91^a^ (0.85)	1.55^b^ (0.61)	1.76^ab^ (0.7)	1.91^a^ (0.94)	5.71^**^
Life satisfaction	3.84^c^ (1.09)	3.95^c^ (1.67)	4.37^b^ (1.18)	5.27^a^ (1.36)	58.79^***^

*Standard deviations are in parentheses. Different letters represent significant difference (p < 0.05)*.

** *p < 0.01 and*

*** *p < 0.001*.

## Discussion

The present study utilized the CMS to explore the combined effect of multiple consumer motivations on well-being. We used LPA as a research method to reveal the patterns of consumer motivation in Chinese culture and identified four consumer motivation profiles: enthusiastic consumers; balanced consumers; rational consumers; and apathetic consumers.

Among the four profiles, rational consumers had not previously been identified. Consumers in this group are concerned with saving money as well as comfort, ethics, and quality. Previous research supports the idea that the goal of saving money should be distinguished from the goal of increasing value through returns (Barbopoulos and Johansson, 2017). The rational consumer seems to be motivated by dual goals: not merely lower prices, but also to gain returns. Indeed, it is even possible that rational consumers are willing to sacrifice some of their economic benefits for higher levels of comfort, ethics, or quality.

It is noteworthy that the profiles we found have been labeled with similar names in other countries. However, there are differences in the implications of these profiles since they are not based on exactly the same motivational dimensions (e.g., Kim et al., 2014; Singh, 2018). For example, Kim et al. (2014) found four similar consumer groups among US consumers: involved shoppers, showing high levels of all dimensions; pragmatic shoppers, showing high levels of utilitarian shopping benefits and shopping costs and low levels of hedonic shopping benefits; experiential shoppers, showing moderate levels of hedonic benefits and low levels of utilitarian benefits and costs; and nonchalant shoppers, showing low levels of all dimensions. In general, the taxonomy of consumer motivation in this study is based on more comprehensive dimensions and constructed more precisely to better describe the patterns of consumer motivation.

Across profiles, the Chinese consumers in our study were most motivated by ethics and value for money, followed by comfort, quality, and safety, with less motivation for stimulation or social acceptance. We found that ethics and value for money were the strongest consumer motivations in our Chinese sample, which may be influenced by traditional Chinese culture and the value of frugality (Lin et al., 2013). However, the result showed that Chinese consumers are less influenced by social acceptance than earlier research suggested. Studies examining the influence of culture on purchase decisions found that consumers in collectivist cultures, like China, place more importance on social acceptance than consumers in individualistic cultures (Nayeem, 2012). Collective consumers tend to choose brands and symbolic products that represent their social status and reaffirm their similarity to reference group members in their purchase choices (Tanusree and Mandal, 2021). In a study of automobile purchases, it was found that individualistic consumers are more interested in performance and features than prestige or brand, whereas collectivistic consumers may prefer higher-priced automobiles because of the associated status and prestige factors (Nayeem, 2012). These mixed results may be explained by the impact of the COVID-19 pandemic on consumer preferences, with recent studies showing that utilitarian motivations prevail over hedonism in crises (Vázquez-Martínez et al., 2021), and minimalism and ethical consumption are some of the main trends during such crises (Flatters and Willmott, 2009).

Moreover, the present study provided information about the demographic characteristics of each profile. Consumer motivation profiles showed significant differences in age, education, and consumption context. In terms of age, there are more young people in the enthusiastic consumers, while the balanced consumers are a bit older. In terms of education, the highly educated young people in the sample were more likely to be in the rational group, and the less educated were more likely to be in the apathetic group. In addition, an analysis of the consumption context revealed that the clothing industry has a higher proportion of rational consumers, the accommodation context has more balanced consumers, and the travel context has more apathetic consumers. The results showed no significant difference in consumer profiles between the travel and food contexts, which is inconsistent with the findings of the previous investigation into single context segmentation (Song and Bae, 2017; Gong et al., 2020a). These results provide preliminary evidence that consumers’ preferences differ across contexts and call for a more detailed and intensive investigation of the explanations for these differences.

The result showed that the largest proportion of balanced consumers accounted for almost half of the participants. Coincidentally, it is reported that the largest proportion of Indian consumers is also balanced, with a balanced orientation of hedonistic, materialistic, and utilitarian traits—more than one-third (Singh, 2018). This is not the case in other countries, such as America, with the largest share of pragmatic shoppers (showing high levels of utilitarian motivation and shopping costs and low levels of hedonic motivation), nearly half (Kim et al., 2014). Also, results differ by gender compared to America. In our study, we found no significant differences in consumer motivations by gender. In the US, however, all female consumers were either engaged or experienced, whereas all male consumers were rational or pragmatic (Kim et al., 2014). Proportional distributions and gender in consumer motivations across countries may be related to culture and economics (Kim et al., 2014; Singh, 2018). In line with studies on shopping motivation in China, there were no significant differences in shopping motivation based on income or occupation (Parker and Wenyu, 2019).

Consumers’ subjective well-being differed across the four consumer motivation profiles. The enthusiastic group scored highest on all dimensions of consumer motivation, and correspondingly, they had significantly higher positive affect and life satisfaction compared to the other profiles. Surprisingly, they also had the highest negative effect. A tendency toward maximization has been shown to be associated with negative affect (Desmeules, 2002; Schwartz et al., 2002). There is a possibility that they have higher expectations for the product and are more likely to experience stress, frustration, regret, and trigger more negative effects. Consumers in the balanced group scored uniformly on all dimensions and demonstrated moderate levels of positive affect and life satisfaction. Their scores for negative affect were similar to those of other profiles. Compared to the enthusiastic group, the balanced group was shown to be relatively emotionally stable.

For the rational and apathetic groups, there were no significant differences from each other in positive affect or life satisfaction, and both scores were significantly lower than those of the enthusiastic and balanced groups. However, these two groups had different results in terms of negative effects. The apathetic group showed more negative affect, and they were the least happy group found in this study. Rational consumers have the lowest negative effect among the four groups. Notably, there was a roughly reversed tendency in the dimensions that made up the rational group and the apathetic group. Consumers in the rational group had significantly higher scores on the dimensions of value for money, quality, comfort, and ethics, and they may have a greater inclination toward intrinsic motivation. Apathetic consumers scored higher on the dimensions of security and social acceptance, and they may have a greater inclination toward extrinsic motivation. Rational consumers with an intrinsic tendency reported less negative affect, whereas apathetic consumers with an extrinsic tendency reported more negative affect. This was consistent with the findings of previous studies (Schmuck et al., 2000; Kasser and Ryan, 2016), further validating the positive effect of intrinsic motivation on well-being under the Chinese sample.

### Theoretical Implications

This study extends previous findings by using a higher-order approach—LPA to segment consumers into four profiles based on their consumer motivation and identifies a new type of consumer—rational consumers, which had not previously been identified (Ribeiro Cardoso and Carvalho Pinto, 2010; Kabadayi and Paksoy, 2016; Singh, 2018). The taxonomy in this study is based on a more integrative dimension structure of consumer motivation, extending the established theories of hedonistic and utilitarian motivation (Babin et al., 1994; Barbopoulos and Johansson, 2017). The emphasis placed by participants of all four profiles on the ethics dimension implies the importance of normative motivations and indicates the CSM presents a helpful framework for conceptualizing consumer motivations.

The person-centered approach adopted in this study fills the research gap created by the relationships between different combinations of consumer motivations and their well-being. In particular, this study expands the research on consumer motivations and their subjective well-being, which demonstrated the different roles of different patterns of consumer motivation in predicting various outcomes of well-being, whereas previous studies focused only on a certain kind of consumer motivation. These findings emphasize the importance of combining the effects of consumer motivation in predicting both positive and negative outcomes of subjective well-being, as well as the value of assessing consumer motivation on a multi-dimensional basis.

### Practical Implications

Consumers were segmented into four profiles based on seven consumer motivation dimensions, providing an informative approach to understanding the differentiated needs of different types of consumers. Market segmentation based on consumer motivation has proven to be one of the most valuable marketing tools in business promotion and consumer behavior prediction (Kim et al., 2014). Our approach to segmenting consumers provides information about what consumers are pursuing. This information enables us to identify incentives that different types of customers may pay attention to or not. The enthusiastic group has high expectations in all dimensions; the balanced group values each dimension of a product but has lower expectations; the rational group emphasizes dimensions such as value for money, comfort, ethics, and quality; and the apathetic group has no strong motivation for consumption and places emphasis on the dimensions of security, social acceptance, and stimulation. It can help marketers develop customized marketing plans based on the consumption patterns of different consumer motivation segments (Singh, 2018). Rational consumers, for example, are driven by the dimensions of money value and ethics rather than social acceptance. Alternatively, they may choose fairly inexpensive practices over environmentally friendly products, or they may reduce their consumption.

The study explores the relationship between different subtypes of consumer motivation and well-being, which provides insights and opportunities for individuals concerned with their own well-being and for marketers who are concerned with the affective states of their consumers. For example, we conclude that enthusiastic consumers have high levels of positive affect and life satisfaction, but their emotions can easily be negative as well. In order to make an appropriate choice, consumers are advised to seek professional and objective information whenever possible. Individuals need to carefully evaluate the information provided by marketers, and enthusiastic consumers need to be wary of the adverse effects of bad experiences such as disappointment or regret. Marketing strategies do seem to create pleasant choices, but they should pay particular attention to the impact on consumer expectations. Advertisers should be careful that their information does not lead consumers to have overly positive assumptions about the advertised goods (Desmeules, 2002).

### Limitation and Future Research Direction

This research is not without limitations, which is the direction of future research. Culture has been studied as an important influence on motivation and well-being before (Gong et al., 2020a). This research contributes to the literature on consumer motivation and its relationship with well-being. However, the study’s results are unclear in terms of whether they would apply to other cultures. For example, the people in this Chinese sample were motivated by ethics and value for money, but these motivations might be less common in other cultures. Future research could use the same approach and measurement to explore and compare the profiles of consumer motivation in different cultural contexts.

While this study provides insights into consumer motivation and well-being, the results need to be replicated. The cross-sectional data is not sufficient to infer causal relationships. Longitudinal studies will help us to understand the changing trends in consumer motivations and their impact on consumers’ well-being. Other measurement methods (e.g., observation, in-depth interviews) could reduce methodological artifacts and provide information about consumer motivations under real-world conditions.

## Conclusion

This study provides evidence on different types of consumer motivation among Chinese consumers using the person-centered—LAP, and it also supports that consumers’ subjective well-being differed significantly across the four motivation profiles. Chinese consumers were segmented into four profiles based on seven consumer motivation dimensions, providing a specific and informative approach to understanding the differentiated needs of different types of consumers. The enthusiastic group exhibited the highest levels of positive affect and life satisfaction, but they also displayed the highest levels of negative affect as a result of higher expectations. Balanced participants reported moderate life satisfaction, positive affect, and negative affect. Among the rational group, life satisfaction and positive affect were the lowest, as well as negative affect because of their internal goal tendency (Schmuck et al., 2000; Kasser and Ryan, 2016). Apathetic consumers are more likely to be motivated by extrinsic factors, and they are the least happy. The results of this study enrich our theoretical and practical understanding about the relationship between consumer motivation types and subjective well-being.

## Data Availability Statement

The original contributions presented in the study are included in the article/supplementary material, further inquiries can be directed to the corresponding authors.

## Ethics Statement

The studies involving human participants were reviewed and approved by Central South University Institutional Review Board. The participants provided their written informed consent to participate in this study.

## Author Contributions

JX, YG, and JL contributed to conception and design of the study, performed statistical analysis, and wrote the first draft of the manuscript. XT and SJ collected the data. XT, SJ, and TD contributed to manuscript revision, read, and approved the submitted version. All authors contributed to the article and approved the submitted version.

## Funding

This work was supported by grants from the National Natural Science Foundation of China (Grant/Award Number: 72072185 and 71672195) and the Project of the Research Foundation of Education Bureau of Hunan Province, China (Grant/Award Number: 18B443 and 21B0824).

## Conflict of Interest

The authors declare that the research was conducted in the absence of any commercial or financial relationships that could be construed as a potential conflict of interest.

## Publisher’s Note

All claims expressed in this article are solely those of the authors and do not necessarily represent those of their affiliated organizations, or those of the publisher, the editors and the reviewers. Any product that may be evaluated in this article, or claim that may be made by its manufacturer, is not guaranteed or endorsed by the publisher.

## Footnotes

^1^ http://www.wjx.cn

## References

[ref1] Akram U. Ansari A. R. Fu G. Junaid M. (2020). Feeling hungry? let’s order through mobile! Examining the fast food mobile commerce in China. J. Retail. Consum. Serv. 56:102142. doi: 10.1016/j.jretconser.2020.102142

[ref2] Akram U. Fulop M. T. Tiron-Tudor A. Topor D. I. Capusneanu S. (2021a). Impact of digitalization on Customers’ well-being in the pandemic period: challenges and opportunities for the retail industry. Int. J. Environ. Res. Public Health 18:7533. doi: 10.3390/ijerph18147533, PMID: 34299984 PMC8303578

[ref3] Akram U. Junaid M. Zafar A. U. Li Z. Fan M. (2021b). Online purchase intention in Chinese social commerce platforms: being emotional or rational? J. Retail. Consum. Serv. 63:102669. doi: 10.1016/j.jretconser.2021.102669

[ref4] An D. Jeong B. R. Youn N. (2021). Effects of art consumption on consumer well-being. J. Consum. Aff. 55, 1–18. doi: 10.1111/joca.12429

[ref5] Arnold M. J. Reynolds K. E. (2003). Hedonic shopping motivations. J. Retail. 79, 77–95. doi: 10.1016/s0022-4359(03)00007-1

[ref6] Asparouhov T. Muthen B. (2014). Auxiliary variables in mixture modeling: three-step approaches using Mplus. Struct. Equ. Modeling 21, 329–341. doi: 10.1080/10705511.2014.915181

[ref7] Babin B. J. Darden W. R. Griffin M. (1994). Work and/or fun: measuring hedonic and utilitarian shopping value. J. Consum. Res. 20, 644–656. doi: 10.1086/209376

[ref8] Barbopoulos I. Johansson L.-O. (2016). A multi-dimensional approach to consumer motivation: exploring economic, hedonic, and normative consumption goals. J. Consum. Mark. 33, 75–84. doi: 10.1108/jcm-08-2014-1091

[ref9] Barbopoulos I. Johansson L.-O. (2017). The consumer motivation scale: development of a multi-dimensional and context-sensitive measure of consumption goals. J. Bus. Res. 76, 118–126. doi: 10.1016/j.jbusres.2017.03.012

[ref10] Bello D. C. Etzel M. J. (1985). The role of novelty in the pleasure travel experience. J. Travel Res. 24, 20–26. doi: 10.1177/004728758502400104

[ref11] Callea A. Lo Presti A. Mauno S. Urbini F. (2019). The associations of quantitative/qualitative job insecurity and well-being: The role of self-esteem. Int. J. Stress Manage. 26, 46–56. doi: 10.1037/str0000091

[ref12] Chang C. (2020). How branded videos can inspire consumers and benefit brands: implications for consumers’ subjective well-being. J. Advertising 49, 613–632. doi: 10.1080/00913367.2020.1806153

[ref13] Desmeules R. (2002). The impact of variety on consumer happiness: marketing and the tyranny of freedom. Acad. Mark. Sci. Rev. 12, 1–18.

[ref001] Diener E. Emmons R. A. Larsen R. J. Griffin S. (1985). The satisfaction with life scale. J. Pers. Assess. 49, 71–75. doi: 10.1207/s15327752jpa4901_13 16367493

[ref14] Dodds W. B. Monroe K. B. Grewal D. (1991). The effects of price, brand, and store information on buyers’ product evaluations. J. Marketing Res. 28, 307–319. doi: 10.1177/002224379102800305

[ref15] El Hedhli K. Zourrig H. Chebat J.-C. (2016). Shopping well-being: is it just a matter of pleasure or doing the task? The role of shopper’s gender and self-congruity. J. Retail. Consum. Serv. 31, 1–13. doi: 10.1016/j.jretconser.2016.03.002

[ref16] Flatters P. Willmott M. (2009). Understanding the post-recession consumer. Harvard Bus. Rev. 87, 106–112.

[ref17] Fornell C. Larcker D. F. (1981). Evaluating structural equation models with unobservable variables and measurement error. J. Mark. Res. 18, 375–381. doi: 10.1177/002224378101800312

[ref18] Gilboa S. Vilnai-Yavetz I. (2012). Segmenting multicultural mall visitors: the Israeli case. Mark. Intell. Plan. 30, 608–624. doi: 10.1108/02634501211262582

[ref19] Glendinning R. (1988). The concept of value for money. Int. J. Public Sect. Manag. 1, 42–50. doi: 10.1108/eb002926

[ref20] Gong Y. Li J. Xie J. Tan Y. (2020a). Relationship between types of food choice motives and well-being among young and middle-aged Chinese adults. Int. J. Consum. Stud. 44, 369–378. doi: 10.1111/ijcs.12573

[ref21] Gong Y. Tang X. Xie J. Zhang L. (2020b). Exploring the Nexus Between work-to-family conflict, material rewards parenting and adolescent materialism: evidence from Chinese dual-career families. J. Bus. Ethics 176, 593–607. doi: 10.1007/s10551-020-04681-4

[ref22] Hair J. F. Black W. C. Babin B. J. Anderson R. E. (2010). Multivariate Data Analysis: A Global Perspective. Upper Saddle River (N.J.): Pearson education.

[ref23] Hebb D. O. (1958). The motivating effects of exteroceptive stimulation. Am. Psychol. 13, 109–113. doi: 10.1037/h0048220

[ref24] Hou J. W. Elliott K. (2021). Mobile shopping intensity: consumer demographics and motivations. J. Retail. Consum. Serv. 63:102741. doi: 10.1016/j.jretconser.2021.102741

[ref25] Howard J. Gagné M. Morin A. J. S. Van den Broeck A. (2016). Motivation profiles at work: A self-determination theory approach. J. Vocat. Behav. 95-96, 74–89. doi: 10.1016/j.jvb.2016.07.004

[ref26] Hui B. P. (2022). Prosocial behavior and well-being: shifting from the ‘chicken and egg’ to positive feedback loop. Curr. Opin. Psychol. 44, 231–236. doi: 10.1016/j.copsyc.2021.09.017, PMID: 34749240

[ref27] Hwang K. Kim H. (2018). Are ethical consumers happy? Effects of ethical Consumers’ motivations based on empathy versus self-orientation on their happiness. J. Bus. Ethics 151, 579–598. doi: 10.1007/s10551-016-3236-1

[ref28] Johansson L.-O. Barbopoulos I. Olsson L. E. (2020). Deactivating economic motives in green consumption through social and moral salience. J. Consum. Mark. 37, 247–258. doi: 10.1108/jcm-10-2018-2904

[ref29] Kabadayi S. Paksoy B. (2016). A segmentation of Turkish consumers based on their motives to visit shopping centres. Int. Rev. Retail Distrib. Consum. Res. 26, 456–476. doi: 10.1080/09593969.2016.1157513

[ref30] Kasser T. Ryan R. M. (2016). Further examining the American dream: differential correlates of intrinsic and extrinsic goals. Pers. Soc. Psychol. B. 22, 280–287. doi: 10.1177/0146167296223006

[ref31] Kim Y.-K. Lee M.-Y. Park S.-H. (2014). Shopping value orientation: conceptualization and measurement. J. Bus. Res. 67, 2884–2890. doi: 10.1016/j.jbusres.2012.06.006

[ref32] Kim J. J. Nam M. Kim I. (2018). The effect of trust on value on travel websites: enhancing well-being and word-of-mouth among the elderly. J. Travel Tour. Mark. 36, 76–89. doi: 10.1080/10548408.2018.1494086

[ref33] Li J. Gong Y. Xie J. Tan Y. (2021). Relationship between users’ perceptions of coolness and intention to use digital products: a user-centered approach. Inform. Technol. Peopl. 35, 1346–1363. doi: 10.1108/itp-03-2020-0129

[ref34] Lin L. Xi D. Lueptow R. M. (2013). Public face and private thrift in Chinese consumer behaviour. Int. J. Consum. Stud. 37, 538–545. doi: 10.1111/ijcs.12023

[ref35] Lindenberg S. Steg L. (2007). Normative, gain and hedonic goal frames guiding environmental behavior. J. Soc. Issues 63, 117–137. doi: 10.1111/j.1540-4560.2007.00499.x

[ref36] Mehta R. Sharma K. Swami S. (2013). A typology of Indian hypermarket shoppers based on shopping motivation. Int. J. Retail. Distrib. 42, 40–55. doi: 10.1108/ijrdm-06-2012-0056

[ref37] Moisander J. (2007). Motivational complexity of green consumerism. Int. J. Consum. Stud. 31, 404–409. doi: 10.1111/j.1470-6431.2007.00586.x

[ref38] Morin A. J. S. Boudrias J.-S. Marsh H. W. McInerney D. M. Dagenais-Desmarais V. Madore I.. (2016). Complementary variable- and person-centered approaches to the dimensionality of psychometric constructs: application to psychological wellbeing at work. J. Bus. Psychol. 32, 395–419. doi: 10.1007/s10869-016-9448-7

[ref39] Morin A. J. S. Marsh H. W. (2014). Disentangling shape from level effects in person-centered analyses: An illustration based on university teachers’ multidimensional profiles of effectiveness. Struct. Equ. Modeling 22, 39–59. doi: 10.1080/10705511.2014.919825

[ref40] Nayeem T. (2012). Cultural influences on consumer behaviour. Int. J. Bus. Manag. 7, 78–91. doi: 10.5539/ijbm.v7n21p78

[ref41] Nylund K. L. Asparouhov T. Muthén B. O. (2007). Deciding on the number of classes in latent class analysis and growth mixture modeling: a Monte Carlo simulation study. Struct. Equ. Modeling 14, 535–569. doi: 10.1080/10705510701575396

[ref42] Onwezen M. C. Antonides G. Bartels J. (2013). The norm activation model: An exploration of the functions of anticipated pride and guilt in pro-environmental behaviour. J. Econ. Psychol. 39, 141–153. doi: 10.1016/j.joep.2013.07.005

[ref43] Oral C. Thurner J. Y. (2019). The impact of anti-consumption on consumer well-being. Int. J. Consum. Stud. 43, 277–288. doi: 10.1111/ijcs.12508

[ref44] Ormel J. Lindenberg S. Steverink N. Verbrugge L. M. (1999). Subjective well-being and social production functions. Soc. Indic. Res. 46, 61–90. doi: 10.1023/a:1006907811502

[ref45] Parker C. J. Wenyu L. (2019). What influences Chinese fashion retail? Shopping motivations, demographics and spending. J. Fash. Mark. Manag. 23, 158–175. doi: 10.1108/jfmm-09-2017-0093

[ref46] Qiu L. Zheng X. Wang Y. (2008). Revision of the positive affect and negative affect scale. Chinese J. Appl. Psychol. 14, 249–254.

[ref47] Rezvani Z. Jansson J. Bengtsson M. (2018). Consumer motivations for sustainable consumption: the interaction of gain, normative and hedonic motivations on electric vehicle adoption. Bus. Strateg. Environ. 27, 1272–1283. doi: 10.1002/bse.2074

[ref48] Ribeiro Cardoso P. Carvalho Pinto S. (2010). Hedonic and utilitarian shopping motivations among Portuguese young adult consumers. Int. J. Retail. Distrib. 38, 538–558. doi: 10.1108/09590551011052124

[ref49] Samudro A. Sumarwan U. Simanjuntak M. Yusuf E. Z. (2020). Assessing the effects of perceived quality and perceived value on customer satisfaction. Manag. Sci. Let. 10, 1077–1084. doi: 10.5267/j.msl.2019.11.001

[ref50] Schmuck P. Kasser T. Ryan R. M. (2000). Intrinsic and extrinsic goals: their structure and relationship to well-being in German and U.S. college students. Soc. Indic. Res. 50, 225–241. doi: 10.1023/a:1007084005278

[ref51] Schwartz B. Ward A. Monterosso J. Lyubomirsky S. White K. Lehman D. R. (2002). Maximizing versus satisficing: happiness is a matter of choice. J. Pers. Soc. Psychol. 83, 1178–1197. doi: 10.1037/0022-3514.83.5.1178, PMID: 12416921

[ref52] Shahid S. Paul J. (2021). Intrinsic motivation of luxury consumers in an emerging market. J. Retail. Consum. Serv. 61:102531. doi: 10.1016/j.jretconser.2021.102531

[ref53] Shim K. Cho H. (2021). Latent profile analysis of ethical consumers in the United States and Malaysia. Int. J. Consum. Stud. 46, 249–267. doi: 10.1111/ijcs.12671

[ref54] Singh D. P. (2018). Integration of materialism with shopping motivations: motivations based profile of Indian mall shoppers. J. Asia Bus. Stud. 12, 381–401. doi: 10.1108/Jabs-05-2016-0075

[ref55] Song H. Bae S. Y. (2017). Understanding the travel motivation and patterns of international students in Korea: using the theory of travel career pattern. Asia Pac. J. Tour. Res. 23, 133–145. doi: 10.1080/10941665.2017.1410193

[ref56] Steg L. Bolderdijk J. W. Keizer K. Perlaviciute G. (2014). An integrated framework for encouraging pro-environmental behaviour: The role of values, situational factors and goals. J. Environ. Psychol. 38, 104–115. doi: 10.1016/j.jenvp.2014.01.002

[ref57] Tanusree D. Mandal M. K. (2021). Consumer Happiness: Multiple Perspectives. Singapore: Springer

[ref58] Troebs C. C. Wagner T. Heidemann F. (2018). Transformative retail services: elevating loyalty through customer well-being. J. Retail. Consum. Serv. 45, 198–206. doi: 10.1016/j.jretconser.2018.09.009

[ref59] Vázquez-Martínez U. J. Morales-Mediano J. Leal-Rodríguez A. L. (2021). The impact of the COVID-19 crisis on consumer purchasing motivation and behavior. Eur. Res. Manag. Bus. Ec. 27:100166. doi: 10.1016/j.iedeen.2021.100166

[ref60] Wang M. Hanges P. J. (2010). Latent class procedures: applications to organizational research. Organ. Res. Methods 14, 24–31. doi: 10.1177/1094428110383988

[ref61] Watson D. Clark L. A. Tellegen A. (1988). Development and validation of brief measures of positive and negative affect: The PANAS scales. J. Pers. Soc. Psychol. 54, 1063–1070. doi: 10.1037/0022-3514.54.6.1063, PMID: 3397865

[ref62] Wippler R. Lindenberg S. (1987). “Collective phenomena and rational choice,” in The micro-macro link. ed. Alexander J. C. (California: University of California Press), 135–152.

[ref63] Xiao F. Sun L. (2020). Students’ motivation and affection profiles and their relation to mathematics achievement, persistence, and behaviors. Front. Psychol. 11:533593. doi: 10.3389/fpsyg.2020.533593, PMID: 33519570 PMC7841336

[ref64] Zhong J. Y. Mitchell V.-W. (2010). A mechanism model of the effect of hedonic product consumption on well-being. J. Consum. Psychol. 20, 152–162. doi: 10.1016/j.jcps.2010.01.001

